# Respiratory syncytial virus in pediatric influenza‐like illness cases in Lombardy, Northern Italy, during seven consecutive winter seasons (from 2014–2015 to 2020–2021)

**DOI:** 10.1111/irv.12940

**Published:** 2021-12-17

**Authors:** Laura Pellegrinelli, Cristina Galli, Laura Bubba, Arlinda Seiti, Giovanni Anselmi, Valeria Primache, Lucia Signorini, Serena Delbue, Sandro Binda, Elena Pariani

**Affiliations:** ^1^ Department of Biomedical Sciences for Health University of Milan Milan Italy; ^2^ Department of Biomedical, Surgical and Dental Sciences University of Milan Milan Italy; ^3^ Interuniversity Research Center on Influenza and Other Transmissible Infections (CIRI‐IT), University of Genoa Genoa Italy

**Keywords:** influenza‐like illness, influenza surveillance, respiratory syncytial virus (RSV), risk of infection children

## Abstract

**Introduction:**

Respiratory syncytial virus (RSV) is the major cause of lower respiratory tract illness in young children and can also cause influenza‐like illness (ILI). Here we investigated the epidemiological features of RSV infection in pediatric ILI cases in Lombardy (a region in Northern Italy accounting nearly 10 million inhabitants) from 2014–2015 to 2020–2021 winter seasons.

**Material and Methods:**

Data for this study were retrieved and statistically analyzed from the database of virological influenza surveillance of the regional reference laboratory for Lombardy within the Italian influenza surveillance network (InfluNet).

**Results:**

RSV accounted for nearly 19% of pediatric ILI with a risk of infection nearly two‐fold greater than that of individuals ≥15 years. RSV positivity rate increased to 28% considering 0–5 years old children. Although in children ≤5 years the risk of infection from influenza viruses resulted nearly two‐fold higher than the risk of RSV infection, the age group 4–6 months and 7–12 months showed a five‐fold greater risk of infection from RSV than from influenza. Children ≤5 years of age with pre‐existing underlying health conditions had a nearly five‐fold greater risk of getting RSV infection than otherwise healthy 0–5 years old children. RSV was identified in ILI cases <15 years of age in all considered winter seasons except in the 2020–2021 season.

**Discussion:**

Sentinel surveillance of ILI allowed us to identify groups at higher risk of RSV and influenza infection and to define the start, duration, timing, and intensity of the RSV and influenza community circulation. This surveillance approach can be implemented to assess the RSV circulation and impact in a real‐time manner.

## 1 INTRODUCTION

Respiratory syncytial virus (RSV) has been recognized as the second most common cause of death in infants. It has been estimated that RSV is responsible for 34 million new episodes of lower respiratory tract infection (LRTI) worldwide,^1^ accounting in 2005 for 199 000 deaths among children under 5 years, 99% of which occurred in low‐resource settings. However, also in high income countries, such as the UK, the average rate of hospital admissions due to RSV bronchiolitis has increased by an average of 1.8% per year since 2004^2^ with RSV bronchiolitis accounting for 12% of admissions to pediatric intensive care unit.^2^

RSV is recognized as the pathogen most frequently involved in acute viral bronchiolitis and pneumonia in young children; in fact, in children under 2 years of age it causes from 40% to 90% of bronchiolitis hospitalizations and up to 50% of pneumonia admissions.^3^, ^4^, ^5^ In this age group, the highest frequency of RSV‐related severe manifestations and mortality are generally observed, with a peak in children under 3 months of life.^6^ Moreover, severe outcomes are frequently associated with pre‐existing underlying health conditions, such as chronic lung diseases or congenital heart diseases.^3^, ^7^ The reported RSV hospitalization rates across the European countries ranged from 2.5 to 11 per 1000 children within the first 4 years of life, and from 19 to 22 per 1000 children among those younger than 12 months of age.^8^ These data highlight the significant health, financial and social impact of RSV in both high‐ and low‐income countries.

RSV is an RNA virus of the *Pneumoviridae* family^9^ that primarily spreads via respiratory droplets when a person coughs or sneezes, and through direct contact with contaminated surfaces.^10^ RSV is one of the most contagious human pathogens, with over 80% of children experiencing RSV infections by 2 years of age.^11^ Reinfection occurs throughout life and can occur more than once in the same season.^12^

RSV seasonality is highly dependent on geographic location and climate. In temperate regions an annual seasonal pattern is predictably limited to 3–5 months during winter and autumn.^13^, ^14^ Numerous explanations for this have been proposed, including the possibility that inclement climate modifies human behavior, reducing outdoor activities and increasing indoor crowding enhancing exposure and transmission of RSV^15^, ^16^, ^17^ or that the low temperatures present during winter prolong the stability of RSV in fomites.^14^

Besides hospitalisations and severe manifestations,^18^, ^19^, ^20^, ^21^, ^22^ RSV infections can also lead to mild symptoms causing influenza‐like illness (ILI), clinically undistinguishable from other common respiratory infections.^20^, ^23^, ^24^, ^25^, ^26^ Since RSV infections occur primarily during autumn and winter—thus overlapping the seasonal circulation of influenza viruses^27^—the virological surveillance of ILI can be a useful tool to monitor RSV circulation—in addition to influenza viruses—in the general population so as to define community viral transmission. The primary objective of this study was to analyze the epidemiological characteristics of RSV in pediatric (<15 years of age) ILI cases observed in the framework of the epidemiological and virological surveillance of influenza‐like illness (ILI) in Lombardy (Northern Italy) during seven consecutive winter seasons, namely from 2014–2015 to 2020–2021. The secondary objectives of this study were (i) to describe the epidemiology of RSV infection in children ≤5 years of age with ILI during seven consecutive seasons (from 2014–2015 to 2020–2021), focusing on the following age groups: 0–3 months, 4–6 months, 7–12 months, and 13–24 months and (ii) to compare the epidemiological characteristics of RSV and influenza virus infections in children ≤5 years of age with ILI over the study period.

## 2 METHODS

Data for this report were retrieved from the database of virological influenza surveillance of the regional reference laboratory for Lombardy (Northern Italy), operating within the Italian influenza surveillance network (InfluNet).^28^

### 2.1 Epidemiological and virological surveillance

The Italian influenza surveillance network (InfluNet)^28^ combines epidemiological and virological surveillance of influenza to provide information to monitor influenza activity in Italy. The network aims to track influenza epidemics, detecting their start, monitoring their spatial–temporal spread, identifying populations at risk and circulating influenza viruses, and estimating the impact on the community and healthcare structures.

InfluNet relies on the voluntary participation of sentinel physicians (both pediatricians and general practitioners) who survey 2% of the general population, ensuring the representativeness of all age groups (0–4 years, 5–14 years, 15–64 years, and ≥65 years), with homogenous geographical distribution.^28^ To increase surveillance sensitivity, the percentage of population under surveillance was increased from 2% to 4% in the 2020–2021 season.

All sentinel physicians are involved in epidemiological surveillance and in charge to report weekly on the number of outpatients seeking care in their ambulatory facilities for ILI occurrence.^28^ The “zero reporting” strategy ‐ based on the report of the absence of ILI cases under syndromic surveillance ‐ is adopted. This is crucial for ensuring that sentinel physicians have not merely forgotten to report cases. A number of sentinel physicians is also in charge of collecting respiratory samples (nasopharyngeal swabs, NPS) for virological surveillance.^28^

Virological surveillance has an observation period of 28 weeks and extends from week 46 to week 17 of the following year, according to the InfluNet operational protocol.^29^ Sampling must be performed in the acute phase of illness and within 7 days from symptoms' onset.

### 2.2 Case definition

The ILI case definition according to the European Center for Disease Prevention and Control (ECDC)^30^ is: an abrupt onset of fever (>38°C) or feverishness, one or more respiratory symptoms (cough, sore throat and/or shortness of breath) and one or more systemic symptoms (myalgia, headache and/or malaise). For children, the case definition of ILI also includes: irritability, loss of appetite and persistent, inconsolable crying. Influenza in toddler is usually associated with vomit and diarrhea and sometimes with fever. Among pre‐schooling children influenza infection can cause bloodshot eyes and conjunctivitis with fever and among children aged 1–5 years, laryngo‐tracheitis and bronchitis/bronchiolitis should be considered.

### 2.3 Surveillance data

The following epidemiological surveillance data were collected through the InfluNet database (www.iss.it/site/rmi/influnet):

- Aggregate consultations for ILI by week and by age group reported by sentinel primary health care providers in Lombardy.
- Case‐based data on:
  ○ demographic characteristics: age and gender (male/female)
  ○ clinical characteristics:
    - date of symptoms onset
    - date of NPS collection
    - presence/absence of pre‐existing underlying health conditions (i.e., cardiovascular diseases, chronic respiratory diseases, metabolic diseases, immunodeficiencies)
    - influenza vaccination status
    - administration of antiviral influenza drugs.

The following virological influenza surveillance data are collected through the database of the regional reference laboratory:

- types and subtypes of influenza viruses collected from sentinel sources
- detection of RSV.

This analysis considered data from seven consecutive winter seasons, namely, from 2014–2015 to 2020–2021.

### 2.4 Statistical analysis

Descriptive and quantitative analyzes were conducted using Microsoft Excel 2010, Open Epi (version 3.01), R statistical computing software (version 3.3.1) and STATA (version 13).

The frequency of positive NPSs was expressed as crude proportion with corresponding 95% confidence interval (95% CI) calculated by Mid‐P exact test assuming a normal distribution. Proportions between sub‐groups (i.e., age groups and presence of comorbidities) were compared using the Chi‐square test based on binomial distribution.

The risk of infection was expressed as the number of individuals with RSV or influenza laboratory‐confirmed infection out of the total number of individuals with ILI. The conditional maximum‐likelihood estimate (CMLE) of odds ratios (OR) with corresponding 95% CI was calculated.

For continuous variables, such as age distribution, the unpaired *t* test was performed and the inter‐quartile range (IQR) was computed as difference of first and third quartile.

We estimated influenza and RSV seasonal characteristics, including season onset (or start), duration, peak and offset (or end) applying the RS10 method, which defines the start of epidemic season as the first 2 consecutive weeks when virus detection exceeds 10% of virus‐positivity rate.^31^

A *p* value <0.05 was considered significant (two‐tailed test).

## 3 RESULTS

### 3.1 Epidemiological and virological results of influenza‐like illness surveillance

**FIGURE 1:**
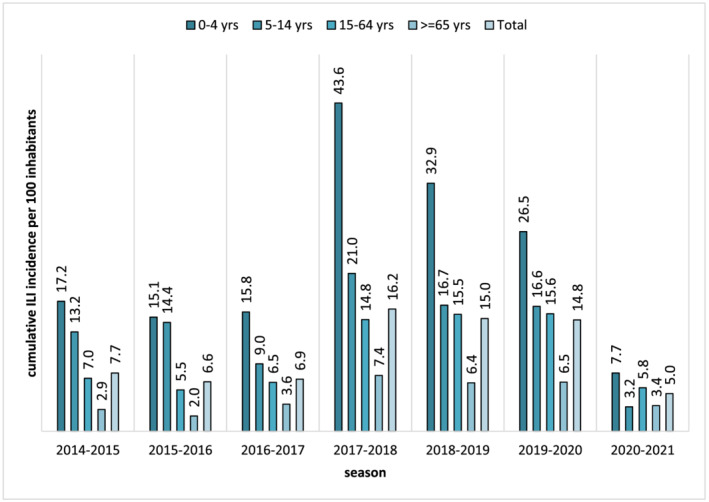
Total and age group cumulative incidence of influenza‐like illness (ILI) cases (per 100 inhabitants) by season (from 2014–2015 to 2020–2021 winter season) in Lombardy

During the seven winter seasons considered (from 2014–2015 to 2020–2021), the cumulative incidence of ILI cases in the general population ranged from 5% in 2020–2021 to 16.2% in 2017–2018, with a mean value of 10.3% (Figure 1). In all seasons the highest cumulative incidence of ILI cases was reported in the 0–4 years age group (mean: 22.7%, range: 7.7%–43.6%), followed by the 5–14 years age group (mean: 13.4%, range: 3.2%–21.0%), then decreasing in the adult (15–64 years) age group (mean: 10.1%, range: 5.5%–15.6%), and in the ≥65 years age group (mean: 4.6%, range: 2.0%–7.4%) (Figure 1).

From 2014–2015 to 2020–2021 season, 3971 NPS were collected from as many ILI outpatients (mean number of NPS/season: 567, range: 519 [in 2020–2021] – 631 [in 2019–2020]); males accounted for 51.0% (2024/3971; *p* = 0.6) of ILI cases; the median age was 34 years (IQR: 43.4 years) and 23.7% (943/3971) of ILI cases reported the presence of comorbidities.

**FIGURE 2:**
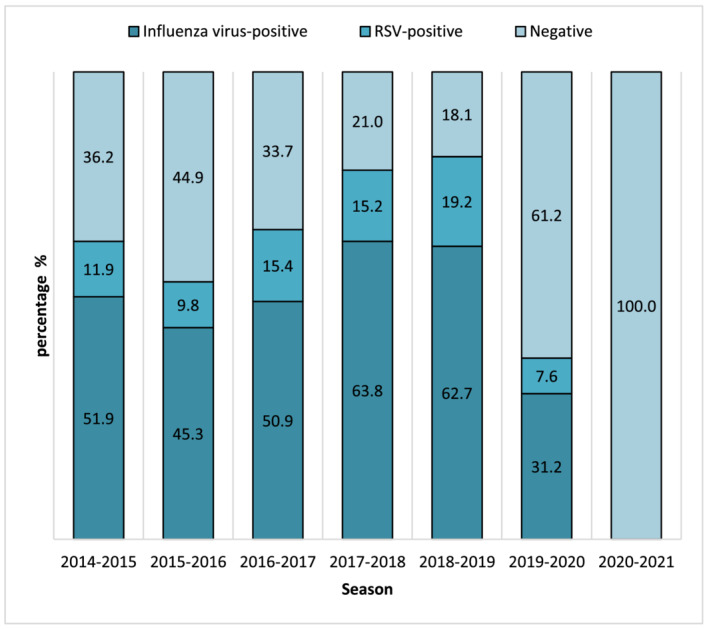
Percentages of nasopharyngeal swabs (NPSs) that resulted positive to influenza virus or respiratory syncytial virus (RSV) by season (from 2014–2015 to 2020–2021 winter season) in Lombardy

The percentages of NPSs that resulted positive to influenza virus and RSV by season are reported in Figure 2. Excluding the 2020–2021 winter season when no influenza viruses or RSV were detected, the mean positivity rate for influenza virus was 51.0% (ranging from 31.2% in 2019–2020 to 63.8% in 2017–2018) whereas for RSV it was 13.2% (ranging from 7.6% in 2019–2020 to 19.2% in 2018–2019).

From 2014–2015 to 2020–2021 season, 44.2% (1755/3971) of NPSs collected from ILI cases resulted positive to influenza virus detection. Of these influenza virus‐positive cases, males accounted for 51% (895/1755; *p* = 0.6); the median age was 28 years (IQR: 42.3 years); the presence of comorbidities was recorded in 22.5% (394/1755) of influenza virus‐positive cases, with no differences among seasons.

From 2014–2015 to 2020–2021 influenza season, 2362 NPSs (59.4%) collected from ILI cases were tested for RSV, resulting in a RSV positivity rate of 9.5% (224/2362). Males accounted for 52.7% (118/224; *p* = 0.6); the median age was 5 years (IQR: 43 years); the presence of underlying medical conditions was recorded in 21.4% (48/224) of RSV‐positive ILI cases, with no differences among seasons.

**FIGURE 3:**
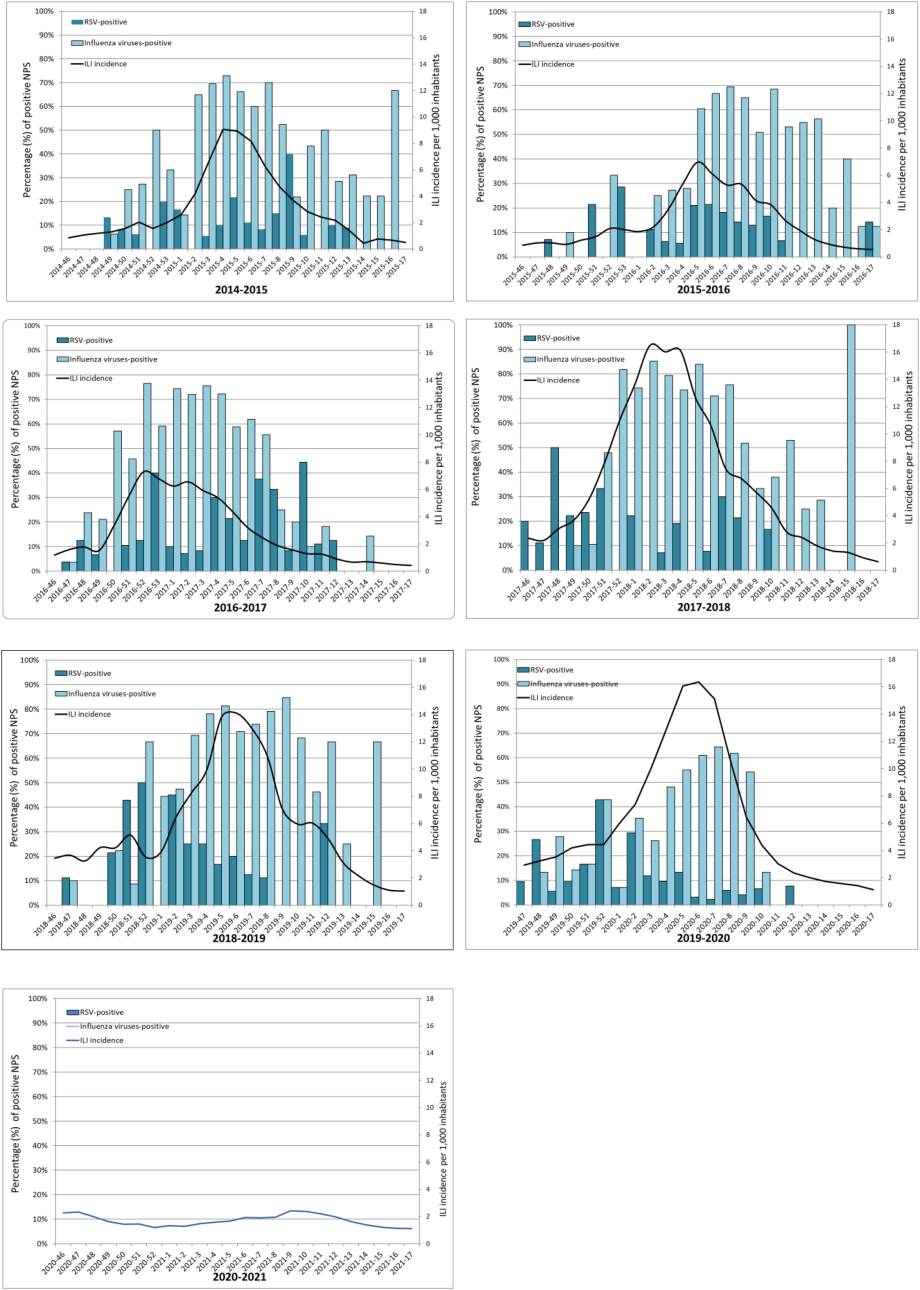
Percentages of respiratory syncytial virus (RSV)‐positive and influenza virus‐positive nasopharyngeal swabs (NPSs) by week and weekly incidence of influenza‐like illness (ILI) cases (per 1000 inhabitants) in Lombardy by season (from 2014–2015 to 2020–2021)

The percentage of influenza virus‐ and RSV‐positive NPSs by week along with the weekly incidence of ILI cases (per 1000 inhabitants) in Lombardy by season (from 2014–2015 to 2020–2021 season) are presented in Figure 3. The weekly distribution of laboratory‐confirmed influenza cases almost overlapped the weekly distribution of ILI cases in all seasons, whereas the distribution of RSV‐positive cases changed season by season (Figure 3). RSV‐positive rate exceeded 10% of ILI cases before the ILI peak in three out of six seasons (namely, in 2017–2018, 2018–2019, 2019–2020), during the ILI peak in two seasons (2014–2015, 2015–2016) and after the ILI peak in one season (2016–2017).

Table 1 summarizes the characteristics of ILI, influenza and RSV epidemic by season. Influenza epidemic onset ranged from week 48 to week 52, the week of peak ranged from week 2 to week 7 and the epidemic offset ranged from week 10 to week 15. According to the RS10 method, the average length of influenza epidemics was 15 weeks, with the shortest duration of influenza epidemic in 2015–2016 (12 weeks) and the longest in 2014–2015 season (19 weeks).

**TABLE 1:** Characteristics of RSV and influenza virus (IV) epidemic in ILI cases from 2014–2015 to 2020–2021 winter seasons in Lombardy

Season	Week of onset			Week of peak			Week of offset			Epidemic width (N. of weeks)		
	ILI	RSV	IV	ILI	RSV	IV	ILI	RSV	IV	ILI	RSV	IV
2014‐2015	50‐2014	4‐2015	50‐2014	4‐2015	9‐2015	4‐2015	12‐2015	12‐2015	15‐2015	16	9	19
2015‐2016	52‐2015	5‐2016	52‐2015	5‐2016	6‐2016	7‐2016	11‐2016	10‐2016	10‐2016	14	5	12
2016‐2017	49‐2016	51‐2016	48‐2016	53‐2016	10‐2017	3‐2017	7‐2017	12‐2017	11‐2017	11	15	17
2017‐2018	46‐2017	46‐2017	50‐2017	2‐2018	7‐2018	2‐2018	12‐2018	8‐2018	13‐2018	19	15	16
2018‐2019	45‐2018	50‐2018	52‐2018	6‐2019	2‐2019	2‐2019	14‐2019	8‐2019	13‐2019	22	11	16
2019‐2020	46‐2019	47‐2019	48‐2019	6‐2020	52‐2019	7‐2020	12‐2020	5‐2020	10‐2020	20	11	15
2020‐2021	42‐2020	n.d.	n.d.	n.d.	n.d.	n.d.	47‐2020	n.d.	n.d.	6	n.d.	n.d.

*Note*: For each season, the week of onset (or start), peak, offset (or end), and epidemic width (duration) of ILI and RSV/IV epidemic are reported. The season onset, peak, offset, and epidemic width were estimated applying the RS10 method, which define the start of RSV and influenza epidemic season as the first two consecutive weeks when virus detection exceeds 10% of RSV/IV‐positivity rate. The onset of ILI corresponds to the week when ILI incidence exceeds the threshold value of 2‰. n.d.: not determined.

RSV epidemic onset ranged from week 46 to week 5, with a peak of RSV detection from week 52 to week 10, and epidemic offset ranged from week 5 to week 12 (Table 1). According to the RS10 method, the average length of RSV epidemic was 11 weeks, with the shortest duration observed in 2015–2016 (5 weeks) and the longest in 2016–2017 and 2017–2018 seasons (15 weeks).

### 3.2 Respiratory syncytial virus epidemiology and circulation in pediatric influenza‐like illness (<15 years old)

Overall, 31.7% (750/2362) of NPSs tested for RSV were collected from pediatric (i.e., <15 years old) ILI outpatients. Their characteristics are detailed in Table 2. Males accounted for 56.1% (421/750; *p* = 0.2). The median age was 5 years (IQR: 8 years) with no differences among the seven winter seasons; 8.8% (66/750) of ILI cases <15 years reported the presence of comorbidities.

**TABLE 2:** Characteristics of ILI cases <15 years old and RSV‐positive cases and distribution by age group and by season (from 2014–2015 to 2020–2021) in Lombardy (n.d.: not determined)

	ILI cases	RSV‐positive cases
No. of cases	750	142
% (95% CI)	100%	18.9% (16.3%–21.9%)
No. of males	421	80
% (95% CI)	56.1% (52.6%–59.6%)	56.3% (48.1%–64.2%)
Median age	5	3
IQR [range] (years)	8 [2–10]	3 [2–5]
No. of cases with comorbidities	66	15
% (95% CI)	8.8% (7.0%–11.0%)	22.7% (14.3%–34.2%)
No. of cases by age group % (95% CI)		
0–5 years	370	103
	49.4% (45.7%–52.9%)	27.8% (23.5%–32.6%)
6–10 years	227	28
	30.2% (27.0%–33.6%)	12.3% (8.7%–17.2%)
11–14 years	153	11
	20.4% (17.6%–23.5%)	7.2% (4.0%–12.4%)
No. of cases by season % (95% CI)		
2014–2015	83	22
	11.1% (9.0%–13.5%)	26.5% (18.2%–36.9%)
2015–2016	101	13
	13.5% (11.2%–16.1%)	12.9% (7.7%–20.8%)
2016–2017	77	24
	10.3% (8.3%–12.6%)	31.2% (21.9%–42.2%)
2017–2018	80	22
	10.7% (8.6%–13.1%)	27.5% (18.9%–38.1%)
2018–2019	90	27
	12.0% (9.9%–14.5%)	30.0% (21.5%–40.1%)
2019–2020	232	34
	30.9% (27.7%–34.4%)	14.7% (10.7%–19.8%)
2020–2021	87	0
	11.6% (10.2%–15.0%)	0% (n.d.)

Cumulatively, 49.3% (370/750) of these ILI cases were outpatients aged 0–5 years, 30.3% (*n* = 227) belonged to the 6–10 years age group, and 20.4% (*n* = 153) to the 11–14 years age group (Table 2). The number of ILI cases <15 years in the 2019–2020 season was statistically higher (*p* < 0.05) than the number of ILI cases <15 years observed in the other six seasons.

The overall RSV positivity rate was 18.9% (142/750) in ILI outpatients <15 years, accounting for 63.4% (142/224) of all RSV detected in NPSs collected from the complete ILI series from 2014–2015 to 2020–2021 winter season in Lombardy.

56.3% (80/142) of RSV‐positive children were males (*p* = 0.01), making the risk of infection from RSV in males 1.6‐fold (95% CI: 1.0–2.7) greater than that observed among females.

Among ILI cases <15 years, a statistical difference in age distribution between RSV‐positive and RSV‐negative was observed: the median age of RSV‐positive cases was 3 years (IQR: 3 years) and 6 years (IQR: 7 years), respectively (*p* < 0.001). In detail, the highest RSV positivity rate was observed among children 0–5 years (p < 0.001); in fact, RSV positivity rate in 0–5 years ILI outpatients was 27.8% (103/370), 12.3% (28/227) in the 6–10 years age group, and 7.2% (11/153) in the 11–14 years age group (Table 2).

Overall, the risk of infection from RSV in children 0–14 years was nearly two‐fold (OR: 1.9; 95% CI: 1.6–2.4) higher than that observed in individuals ≥15 years of age, and the risk of RSV infection in children 0–5 years was 23‐fold greater (95% CI: 21.9–37.5) than that in 6–14 years children. In detail, the risk of infection from RSV dramatically increased among the youngest: in fact, the risk of infection from RSV in the 0–5 years age group resulted 18.3‐fold (95% CI: 11.7–29.3) greater than that observed in the 6–10 years age group, 33.2‐fold (95% CI: 17.8–66.9) higher than that in the 11–14 years age group, and seven‐fold higher (95% CI: 5.1–9.7) than that observed among ILI cases ≥15 years (Table 3). The risk of infection from RSV in the age group 6–10 years resulted 89.6‐fold (95% CI: 44.4–194) greater than that observed in the 11–14 years age group (Table 3).

**TABLE 3:** Risk of RSV infection ‐ expressed as odds ratios with corresponding 95% CI—in ILI cases by age group from 2014–2015 to 2020–2021 winter seasons in Lombardy

Age group	6–10 years	11–14 years	≥15 years
0–5 years	**18.3 (11.7–29.3)**	**33.2 (17.8–66.9)**	**7.0 (5.1–9.7)**
6–10 years	—	**89.6 (44.4–194.0)**	**2.5 (1.6–4.0)**
11–14 years	—	—	1.4 (0.7–2.6)
0–14 years	—	—	**1.9 (1.6–2.4)**

*Note*: Significant ORs are in bold.

No significant difference in RSV positivity rate was observed among ILI cases <15 years with or without comorbidities (22.7% vs. 18.5%; *p* = 0.4) (Table 2).

RSV was identified in NPSs collected from ILI 0–14 years of age in all winter seasons except in the 2020–2021 season. The mean RSV positivity rate in ILI outpatients ≤15 years of age from 2014–2015 to 2019–2020 seasons was 23.8%; the RSV positivity rate by season ranged between 12.9% (13/101) in 2015–2016 and 31.2% (24/77) in 2016–2017 (Table 2). Considering only the seasons with RSV circulation, a lower (p < 0.05) frequency of RSV detection was observed in 2015–2016 compared to 2014–2015, 2016–2017 and 2017–2018 season.

### 3.3 Epidemiology of respiratory syncytial virus in children ≤5 years of age with ILI

A total of 370 ILI cases ≤5 years were tested for RSV, information on gender, age and presence of comorbidities are detailed in Table 4. Males accounted for 56.5% (*p* = 0.3) of ILIs ≤5 years of age; the median age was 28 months (IQR: 26.8 months) with no differences among considered winter seasons. Overall, 5.1% (19/370) of ILI cases ≤5 years were children with comorbidities, 52.6% (10/19) of whom were outpatients aged 0–2 years.

**TABLE 4:** Characteristics of all ILI cases ≤5 years of age and RSV‐positive cases and distribution by age group and by season (from 2014–2015 to 2020–2021) in Lombardy (n.d.: not determined)

	ILI cases	RSV‐positive cases
No. of cases	370	103
% (95% CI)	100%	27.8% (23.5–32.6%)
No. of males	209	61
% (95% CI)	56.5% (51.4–61.4%)	59.2% (49.6–68.2%)
Median age	28	27
IQR [range] (months)	26.8 [43.9–17.1]	24.9 [45.6–17.1]
No. of cases with comorbidities	19	11
% (95% CI)	5.1% (3.3–7.9%)	57.9% (36.3–76.9%)
No. of cases by age group % (95% CI)		
0–3 months	5	0
	1.4% (0.6–3.1%)	0% (n.d.)
4–6 months	11	4
	2.9% (1.7–5.2%)	36.4% (15.2–64.6%)
7–12 months	48	14
	13% (9.9–16.8%)	29.2% (18.2–43.2%)
13–24 months	105	29
	28.4% (24.0–33.2%)	27.6% (20–36.9%)
25–60 months	201	56
	54.3% (49.2–59.3%)	27.9% (22.1–34.4%)
No. of cases by season % (95% CI)		
2014–2015	45	16
	12.2% (9.2–15.9%)	35.6% (23.2–50.2%)
2015–2016	59	10
	15.9% (12.6–20.0%)	16.9% (9.5–28.5%)
2016–2017	38	12
	10.3% (7.6–13.8%)	31.6% (19.1–47.5%)
2017–2018	39	13
	10.5% (7.8–14.0%)	33.3% (20.6–49.0%)
2018–2019	53	21
	14.3% (11.1–18.3%)	39.6% (27.6–53.1%)
2019–2020	99	31
	26.8% (22.5–31.6%)	31.3% (23.0–41.0%)
2020–2021	37	0
	10% (7.3–13.5%)	0% (n.d.)

Cumulatively, 45.7% (169/370) of ILI cases ≤5 years were outpatients aged 0–2 years: 1.4% (*n* = 5) belonged to the 0–3 months age group, 2.9% (*n* = 11) to the 4–6 months age group, 13% (*n* = 48) to the 7–12 months age group, 28.4% (*n* = 105) to the 13–24 months age group; 54.3% (*n* = 201) of ILI cases were children belonging to the 25–60 months age group (Table 4).

27.8% (*n* = 103) of NPSs collected from 0–5 years ILI cases resulted positive to RSV, accounting for 46% (103/224) of all RSV detected in Lombardy during the study period.

59.2% (61/103) of RSV‐positive children were males (*p* = 0.004), making the risk of infection from RSV 2.1‐fold (95% CI: 1.2–2.6) greater in males than that in females.

Within the 0–5 years age group, RSV‐positive and RSV‐negative cases had a similar age, with a median age of 27 months (IQR: 24.9 months) and 28 months (IQR: 28.4 months), respectively (*p* = 0.2).

54.3% (56/103) of RSV‐positive samples were collected from children belonging to the 25–60 months age group, whereas 45.7% (47/103) of RSV‐positive samples were collected from children ≤24 months of age. In particular, among children ≤24 months of age, RSV positivity rate was 36.4% (4/11) in the 4–6 months age group, 29.2% (14/48) in the 7–12 months age group, and 27.6% (29/105) in the 13–24 months age group. No RSV (0/5) was identified in NPSs collected from children ≤3 months (Table 4).

As shown in Table 5, the risk of infection from RSV increased among the youngest. In fact, the risk of RSV infection in the 4–6 months, 7–12 months and 12–24 months age groups was 5.5‐fold (95% CI: 1.4–19.9), 4‐fold (95% CI: 1.9–8.1), and 3.7‐fold (95% CI: 2.1–6.4) greater than that observed in children aged 6–14 years, respectively. The risk of infection from RSV in the 25–60 months age group resulted 3.7‐fold (95% CI: 2.4–5.9) higher than that observed in children belonging to the 6–14 years age group (Table 5). However, no differences in the risk of infection from RSV were identified comparing the age groups of 4–6 months, 7–12 months, 12–24 months and 24–60 months (Table 5).

**TABLE 5:** Risk of RSV infection—expressed as odds ratios with corresponding 95% CI—in ILI cases by age group from 2014–2015 to 2020–2021 winter seasons in Lombardy

Age group	7–12 months	13–24 months	25–60 months	6–14 years
4–6 months	1.4 (0.3–5‐6)	1.5 (0.3–5.5)	1.4 (0.4–5.3)	**5.5 (1.4–19.9)**
7–12 months	—	1.0 (0.5–2‐3)	1.0 (0.5–2‐1)	**4.0 (1.9–8.1)**
13–24 months	—	—	0.9 (0.5–1.7)	**3.7 (2.1–6.4)**
25–60 months	—	—	—	**3.7 (2.4–5.9)**

*Note*: Significant ORs are in bold.

Among ILI cases ≤5 years of age and with comorbidities, the RSV positivity rate was 40.7% (11/27), resulting in a 4.6‐fold (95% CI: 2.4–9.0) greater risk of RSV infection than that observed in ILIs ≤5 years of age without comorbidities (26.9%; 92/342).

RSV was identified in NPSs collected from ILI 0–5 years of age in all winter seasons except in the 2020–2021. The mean RSV positivity rate in ILI outpatients aged 0–5 years from 2014–2015 to 2019–2020 winter seasons was 26.9%. The RSV positivity rate by season ranged between 16.9% (10/49) in 2015–2016 and 39.6% (21/53) in 2018–2019 season (Table 4). Considering the seasons with RSV circulation, in the 2015–2016 a lower percentage (*p* < 0.001) of RSV‐positive NPSs was observed compared to 2014–2015, 2016–2017, 2017–2018, and 2018–2019 seasons (Table 4).

### 3.4 Comparison between respiratory syncytial virus‐positive and influenza virus‐positive influenza‐like illness cases ≤5 years of age

Overall, in ILI cases ≤5 years of age, the risk of infection from influenza virus resulted nearly two‐fold (OR: 1.8; 95% CI: 1.4–2.4) greater than the risk of infection from RSV.

Among ILI children 0–5 years of age, RSV‐positive cases were significantly younger than influenza virus‐positive cases (2.3 years vs. 4.0 years; *p* < 0.001).

In the 4–6 months age group the risk of infection from RSV was 6.4‐fold (95% CI: 1.6–29) higher than the risk of infection from influenza virus; a similar picture was observed also among 7–12 months old age group where the risk of RSV infection resulted 4.9‐fold (95% CI: 1.5–18.3) higher than the risk of influenza virus infection.

Among ILI cases ≤5 years of age with comorbidities, the risk of RSV infection was 19‐fold (95% CI: 10.6–36) higher than the risk of influenza virus infection.

## 4 DISCUSSION AND CONCLUSIONS

These results demonstrate that RSV significantly contributes to ILI cases in children <15 years accounting for nearly 19% of all cases, and particularly in children under 5 years of age where the RSV positivity rate reached up to 28%. More than one‐fourth (27.6%) of RSV infections occurred in children in their second year of life (13–24 months of age).

Despite the relatively small sample size of ILI under 5 years of age analyzed, the risk of infection from RSV in the 0–5 years age group resulted more than 18‐fold greater than that observed in the 6–10 years age group, and 33‐fold greater than that in the 11–14 years age, demonstrating the massive impact of RSV infection in young children.

Moreover, children ≤5 years of age with pre‐existing underlying health conditions (such as cardiovascular diseases, chronic respiratory diseases, metabolic diseases, immunodeficiencies) had a nearly five‐fold greater risk of getting RSV infection than otherwise healthy 0–5 years old children.

Although in children ≤5 years the risk of infection from influenza viruses resulted nearly two‐fold higher than the risk of RSV infection, the age group 4–6 months and 7–12 months showed five‐fold greater risk of infection from RSV than from influenza virus.

RSV caused epidemic in all considered seasons—without a specific timing during each season—with the exception of 2020–2021 season. This latter season was characterized by the absence of RSV and influenza viruses circulation in all age groups as a consequence of the non‐pharmaceutical interventions put in place in response to SARS‐CoV‐2 pandemic.^32^ The absence of RSV circulation in the last 2020–2021 season may result in wide cohorts of young children naïve to RSV in the next years, thus potentially causing outbreak of RSV infection with unexpected epidemiological pattern. This study has a number of drawback. The dataset analyzed is that of the epidemiological and virological surveillance of influenza and the case definition of ILI is tailor‐made to catch influenza cases and can be less sensitive to capture RSV cases. The number of ILI cases under 1 year of age, and particularly those under 3 months of age, is very limited, probably because, in case of respiratory infections, parents of neonates and infants seek help from hospital emergency room rather than from pediatric ambulatories. Moreover, the virological surveillance is carried out during the winter time and thus no data on virus circulation is available in the inter‐seasonal periods. Lastly, no specific information on the type of comorbidities is routinely collected in the framework of influenza surveillance thus limiting the granularity of data.

In conclusion, sentinel surveillance of ILI revealed to be a valuable tool to define RSV community circulation and to identify groups at higher risk of RSV infection. The extension of virological surveillance to RSV and other respiratory viruses should be implemented within ILI surveillance to assess their circulation and impact in a real‐time manner.

## AUTHOR CONTRIBUTIONS

**Laura Pellegrinelli:** Conceptualization; data curation; investigation. **Cristina Galli:** Data curation; investigation. **Laura Bubba:** Data curation; methodology. **Arlinda Seiti:** Investigation. **Giovanni Anselmi:** Investigation; methodology. **Valeria Primache:** Investigation; methodology. **Lucia Signorini:** Investigation; visualization. **Serena Delbue:** Conceptualization. **Sandro Binda:** Data curation. **Elena Pariani:** Conceptualization; methodology; project administration; supervision.

## CONFLICT OF INTEREST

None declared.

## INSTITUTIONAL REVIEW BOARD STATEMENT

The study was conducted according to the guidelines of the Declaration of Helsinki and was performed according to the Institutional Review Board guidelines concerning the use of biological specimens for scientific purposes in compliance with Italian law (art.13 D. Lgs 196/2003). Approval from an ethics committee for virus detection and data publication were not required since data and samples from outpatients with ILI were collected and analyzed anonymously within the National Influenza Surveillance Program.

## INFORMED CONSENT STATEMENT

Informed consent for virus detection and data publication was not required since data and samples from outpatients with ILI were collected and analyzed anonymously within the National Influenza Surveillance Program.

### PEER REVIEW

The peer review history for this article is available at https://publons.com/publon/10.1111/irv.12940.

## Data Availability

The datasets generated for this study are available on request from the corresponding author.
